# Teeth and Flour

**Published:** 1878-11

**Authors:** Ephraim Cutter

**Affiliations:** Cambridge, Mass.


### ARTICLE III.

### *Teeth and Flour.*

BY EPHRAIM CUTTER, M. D., CAMBRIDGE, MASS.

Extract from a lecture delivered September 27th, 1878, before the American Academy of Dental Science, Boston.

There is no doubt that the decay of teeth prevails among flour-eating people, and it is very humiliating to our modern civilization to have it characterized so generally by the occurrence of diseased teeth. Young persons may be seen on the lines of travel with several incisors gone and others decayed. In the report of the Massachusetts State Board of Health, 1877, there are some statistics as to the prevalence of decayed teeth among school children in Middlesex County. Out of 880 children, mostly under 12 years of age, two-thirds were found by the teachers (of course rather incompetent examiners,) to have decayed teeth! Some of the schools were as follows: Warren Lawrence Primary, 113 scholars, 100 with decayed teeth; Plympton Street Primary, 74 scholars, 67 with decayed teeth; Highland Street Primary, 71 scholars, 25 with decayed teeth.

It is very probable, that had these same scholars been subjected to an examination by a committee of this Society, that hardly a child could have been found with perfectly healthy teeth. Now it seems most natural to infer that if an individual were to have sound teeth he should possess them during the first decade of life, and if this period passes off with no sound teeth, all the successive periods must transpire with a similar increased deprivation. Statistics like the above, if they are a fair average of the dental condition of the people of Massachusetts, are astounding, and should arrest the attention of the philanthropic anthropologist.

The question, next naturally arises, whether savage races who are so uncivilized as not to feed upon flour present such an amount of prevalence of decayed teeth. The great Indian historian and painter, Catlin, decants upon the beauty and perfection of the teeth of the North American Savages, and calls particular attention to the fullness and perfection of the dental portion of the unburied dead skull.

In the wanderings of a F. R. C. S., among the Krumen of the Grain Coast, of Western Africa, there is this statement:

" Rev. J. S. Wood's Uncivilized Races of Men." J. B. Burr, Hartford, Conn., 1871, p. 545 : "Odontology has its mysteries. Dentists seem or rather seemed to hold as a theory, that destruction of the enamel involves the loss of the tooth. The Krume hack their mastication with a knife or rough piece of hoop-iron, and find that the sharpening instead of producing caries, acts as a preservative by facilitating the laniatory process. Similarly there are physiologists who attribute the preservation of the negro's teeth to his not drinking anything hotter than blood heat. This is mere guessing. The Arabs swallow their coffee nearly boiling, and the East African will devour his agali or porridge when the temperature would scald the hand. Yet both these races have pearls of teeth, except when they chew lime or tobacco. They always demand rice, that being a necessity with them, and as long as they get their pint and a half per diem, of rice, they can stand unlimited work.

" They cook the rice for themselves in their primitive but effective manner, and feed themselves as turkeys are crammed, seizing large handfulls of rice, squeezing them into balls, and contrive in some mysterious way to swallow them without being choked," p. 546 do.

In this country the inquiry was made of the Indian Commissioner, Mr. Smith, as to the prevalence of decayed teeth among the Indians. His reply was, that during an intimate acquaintance with them for a quarter of a century, he had *rarely* seen a decayed tooth.

Allow me to read a letter lately sent from the Indian Territory in reply to similar inquiries:

OFFICE WICHITA AGENCY, INDIAN TERRITORY,
ANADANKA P. O., July 31st, 1878.

"E. CUTTER.—*Dear Sir*.—Your note of inquiry, of July 10th, is at hand. I have delayed answering sooner, in order to gain further information concerning the subject of your queries. Dr. O. G. Given, Physician to the Kiowa and Camanche Indians, writes:

'I do not remember of seeing a single case of decayed teeth among the children under ten years old, of the Indians on this reservation. Their diet is largely meat. He further says: Close observation might probably discern some cases; I think, however, very rarely—the first or milk teeth loosening and dropping out.'

'About the same may be said in reference to the Indians of this reservation. Our physician has removed the primary teeth occasionally, but does not remember to have ever observed decayed teeth in children under ten years of age, and indeed, decayed teeth are rare among the Indians of any age, and are the exception, not the rule.

Their diet is chiefly animal food. At this season of the year, green corn, melons, and the produce of the garden are freely made use of by them, and of late years the government has furnished them some flour, sugar and coffee which they use to some extent in conjunction with animal food; they are extremely fond of coffee. The meat is broiled or sometimes hung in strips and dried, and afterwards broiled. They seldom, or never partake of food without afterwards thoroughly rinsing the mouth with water.'

Respectfully,

MRS. DR. F. GRINNELL."

Now without inquiring further, the comparison between these two classes of mankind in this respect is decidedly in favor of the savage. Now the civilized having the advantages of a regular supply of food, of comfortable houses, of clothing, of warmth, and of the stimulus of high mental culture, it seems strange that the bony tissue should so suffer. A great difference between the two classes lies in the food ; civilization has adopted as a staple article of food, flour ; and flour is an impoverished food ; impoverished in the very elements that go to make up the teeth, viz. phosphorus in its combinations, nearly three-fourths is withdrawn.

Is it not natural to expect that the bony structures should suffer from this great withdrawal ? for it is a great withdrawal. Seventy five per cent. diminution of anything. Can we realize it ? Cut down prices on your fee table three-fourths ; remove three-quarters of the space from this auditorium ; cut off the same quantity of time from the hour of day light; curtail our bank circulation three-fourths; take away three-fourths of the water supply of a city ; would there not be trouble everywhere ? And why should not the bony tissues suffer in like manner when their food is withdrawn ? We think they do, and that this impoverishment in flour of mineral ingredients does go a great way in explaining the defectiveness of teeth in civilized races, and their perfection among the wild races who have lived but little, if at all, upon flour. Perhaps a little evidence in a contrary direction may throw light upon this. A gentleman of your profession informed the writer, that he filled fourteen cavities in the teeth of his first-born child by the time he was four (4) years of age. He then placed his family upon the use of animal food and the unbolted meals of different grains, and the next child had no retarded dentition, and not a decayed tooth up to the same age ! The teeth which decay are not compacted or knit together with the firmness of the healthy teeth. There seems to be an arrest of perfect development—though what can be more natural than to expect imperfect development and decay when three-fourths of their proper dental food is materially withdrawn from an article of diet more largely employed than any other ? How curious it is to see infants not cutting at all, until they are twice as old as they ought to be ! The good effect of whole grain diet is seen in the second child above alluded to. To be sure it is only *one* case, and must not be made too much of. Still, taken in connection with the evidence from the savage races of man, the conclusion cannot be resisted, that a portion of the present existing dental diseases might be avoided by simply persuading the people to give up the use of a food which the accurate test of the chemist show us to be so lacking of mineral ingredients and consequently so unworthy of the high estimation it has at present.

Another important question is, as to how long time it would take to produce any effect in this direction. Some think it must take a long series of years, generations perhaps. While it is not intended to ignore hereditary or other peculiarities, still it does seem possible, from the case already alluded to—to produce changes faster than this. Severe sickness will, I am told, produce a great deal of physical change for the worse in teeth ; and on the other hand as the general health improves after convalescence, they also increase in firmness and strength—even in the course of six months, showing that the processes of nutrition and repairs in the teeth are not quite so slow as imagined. But be this as it may—let the time required be ever so long, there is no good reason why the laws of healthy eating should not be observed, inculcated and enforced. In the presence of such an audience these views are put forth with some degree of timidity for fear that the speaker may be thought to be carrying coals to New Castle, and only inculcating ideas which the hearers are every day trying to impress upon their patients. Our object in recalling these things is the hope that your body may at sometime be induced in some official manner, to express publicly their opinion of the necessity of a return to articles of diet in which the natural normal proportion of mineral ingredients is preserved. Public opinon is a very hard thing to mould, affect or influence. Only by dentist after dentist, lecture after lecture, society after society, can these truths be so handed in as to wean people from eating flour who love its taste and love its beautiful white color, and will continue to eat it sometime, even if they know its color resembles the paleness of a corpse, and its taste is simply a matter of education and habit.

